# Retinal de novo lipogenesis coordinates neurotrophic signaling to maintain vision

**DOI:** 10.1172/jci.insight.97076

**Published:** 2018-01-11

**Authors:** Rithwick Rajagopal, Sheng Zhang, Xiaochao Wei, Teresa Doggett, Sangeeta Adak, Jennifer Enright, Vaishali Shah, Guoyu Ling, Shiming Chen, Jun Yoshino, Fong-Fu Hsu, Clay F. Semenkovich

**Affiliations:** 1 Department of Ophthalmology and Visual Sciences,; 2 Division of Endocrinology, Metabolism, and Lipid Research,; 3 Division of Geriatrics and Nutritional Science, and; 4 Department of Cell Biology and Physiology, Washington University School of Medicine, St. Louis, Missouri, USA.

**Keywords:** Metabolism, Ophthalmology, Apoptosis, Lipid rafts, Neurodegeneration

## Abstract

Membrane lipid composition is central to the highly specialized functions of neurological tissues. In the retina, abnormal lipid metabolism causes severe forms of blindness, often through poorly understood neuronal cell death. Here, we demonstrate that deleting the de novo lipogenic enzyme fatty acid synthase (FAS) from the neural retina, but not the vascular retina, results in progressive neurodegeneration and blindness with a temporal pattern resembling rodent models of retinitis pigmentosa. Blindness was not rescued by protection from light-evoked activity; by eating a diet enriched in palmitate, the product of the FAS reaction; or by treatment with the PPARα agonist fenofibrate. Vision loss was due to aberrant synaptic structure, blunted responsiveness to glial-derived neurotrophic factor and ciliary neurotrophic factor, and eventual apoptotic cell loss. This progressive neurodegeneration was associated with decreased membrane cholesterol content, as well as loss of discrete n-3 polyunsaturated fatty acid– and saturated fatty acid–containing phospholipid species within specialized membrane microdomains. Neurotrophic signaling was restored by exogenous cholesterol delivery. These findings implicate de novo lipogenesis in neurotrophin-dependent cell survival by maintaining retinal membrane configuration and lipid composition, and they suggest that ongoing lipogenesis may be required to prevent cell death in many forms of retinopathy.

## Abstract 2

Endogenous fatty acid generation is dispensable for retinal development, but required for maintaining microdomain lipid composition that sustains vision through trophic signaling pathways.

## Introduction

Retinal physiology depends on sophisticated signaling by membrane proteins, lipid composition is critical to the function of membrane proteins (1–3), and abnormalities of lipid metabolism contribute to retinal diseases that lead to visual loss (4, 5). Several hereditary retinal diseases are triggered by aberrant ocular lipid metabolism. Mutations in Rab escort protein-1 (*REP-1* or *CHM*), which facilitates geranylgeranyl transfer onto unprenylated Rab GTPases, cause choroideremia — an X-linked retinal degeneration characterized by profound loss of the outer retina and choriocapillaris (6). Mutations in Prominin1 (*PROM1*), which binds cholesterol in plasma membrane microdomains, are associated with retinal macular dystrophy type 2 and Stargardt disease 4 (7, 8). Decreased activity of retinal Elongation of very long–chain fatty acids protein 4 (Elovl4) causes autosomal dominant Stargardt disease 3 (9). Loss of function of ATP binding cassette subfamily a member 4 (ABCA4), which is retina specific and transports retinoids, is associated with both autosomal recessive Stargardt disease 1 and retinitis pigmentosa, a common inherited cause of nyctalopia (night blindness) and photoreceptor loss (10).

Retinitis pigmentosa describes a group of diseases characterized by retinal degeneration caused by mechanisms that are incompletely defined. As with other forms of neurodegeneration, the clinical presentation varies widely and may be influenced by metabolic modifiers such as glucose metabolism (11) and insulin signaling (12). Fatty acid synthase (FAS), which catalyzes the committed step in de novo lipogenesis, is regulated by numerous metabolic modifiers, including glucose and insulin (13). FAS is also highly expressed in neural tissue and important for neurogenesis (14), prompting the question of how FAS regulates neuroretinal function.

There are several determinants of retinal fatty acid composition, including FAS-driven de novo lipogenesis, the synthesis of fatty acids from simple precursors; the Sprecher pathway, the replacement of saturated fatty acids with polyunsaturated fatty acids (PUFAs) through remodeling; and availability of diet-derived essential fatty acids, which reach the retina through poorly understood mechanisms. The relevant contributions of exogenous versus endogenous sources of lipid to normal retinal physiology are unresolved.

FAS generates predominantly palmitate, which may serve as a substrate for numerous downstream biological processes that might contribute to retinal health. Palmitate is abundant in many diets and is transported into the neural retina (15) but, in other tissues, cells can distinguish between newly synthesized and exogenous palmitate (16, 17). We sought to clarify the physiologic roles of de novo lipogenesis in visual function by deleting FAS in the retina.

## Results

### Components of de novo lipogenesis in neural retina.

**Figure 1:**
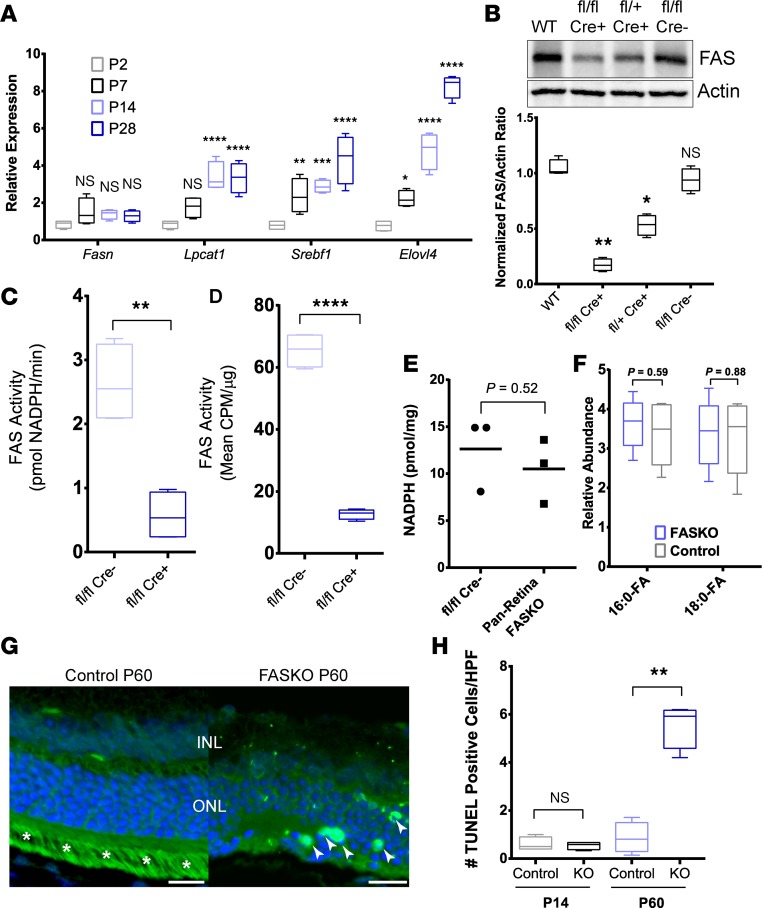
Disruption of FAS activity in the neural retina of FASKO mice. (**A**) Retinal message levels at various developmental stages for FAS and other lipid biosynthetic mediators (*n* = 4 animals/group, 2-way ANOVA). (**B**) FAS protein levels in homozygotes (column 2) and heterozygotes (column 3) compared with controls (columns 1 and 4) with quantitation below (*n* = 4/group, 1-way ANOVA). (**C**) FAS enzyme activity by NADPH consumption (*n* = 4 animals/group, 2-tailed *t* test). (**D**) FAS enzyme activity by incorporation of radiolabel (*n* = 4 animals/group, 2-tailed *t* test). (**E**) Total NADPH levels in whole retinal lysates (*n* = 3/group, 2-tailed *t* test). (**F**) Levels of free palmitate and stearate in retina (*n* = 6/group, 2-tailed *t* test). (**G**) Representative TUNEL staining of FAS-deficient retina and control retina (asterisks show nonspecific staining of outer segments, arrowheads show examples of TUNEL-positive nuclei included for analysis). Size bars: 12.5 μm. (**H**) Quantification of TUNEL staining (*n* = 4 animals/group, 1-way ANOVA). *P < 0.05, **P < 0.01, ***P < 0.001, ****P < 0.0001.

FAS mRNA expression was constitutive throughout retinal development (Figure 1A). The FAS expression pattern was distinctly different from those of message levels for the lipogenic genes lysophosphatidylcholine acyltransferase 1 (*Lpcat1*), sterol regulatory element-binding protein 1 (*Srebf1*), and *Elovl4*, all of which increased over the time course of retinal maturation (Figure 1A).

### Disruption of Fasn increases apoptosis in the neural retina.

To investigate the effects of FAS on neuroretinal physiology, we created mice harboring floxed *Fasn* alleles (16) and Cre recombinase driven by the pan-retinal Chx10 promoter (18). Retinas from mice with homozygous deletion (FASKO) displayed reduced FAS protein, and this reduction was intermediate in heterozygotes compared with retinas from WT mice or floxed mice lacking the Cre recombinase (Figure 1B). Retinal FAS enzyme activity assayed by NADPH consumption (Figure 1C) or radiolabeled palmitate generation (Figure 1D) was markedly reduced in FASKO mice compared with controls. Since the Chx10 promoter is not expected to be active in endothelial cells or microglia, some residual protein expression and enzyme activity were detected in the pan-retinal FASKO tissues (Figure 1, B–D). Despite reduced FAS enzyme activity in the KO tissue, steady-state levels of NADPH, palmitate (16:0-FA), and stearate (18:0-FA) were comparable with control tissues (Figure 1, E and F). FAS deficiency was associated with progressive apoptotic death within the neural retina (Figure 1, G and H).

### FAS is dispensable for retinal development, but its deficiency is associated with neurodegeneration in the mature retina.

**Figure 2:**
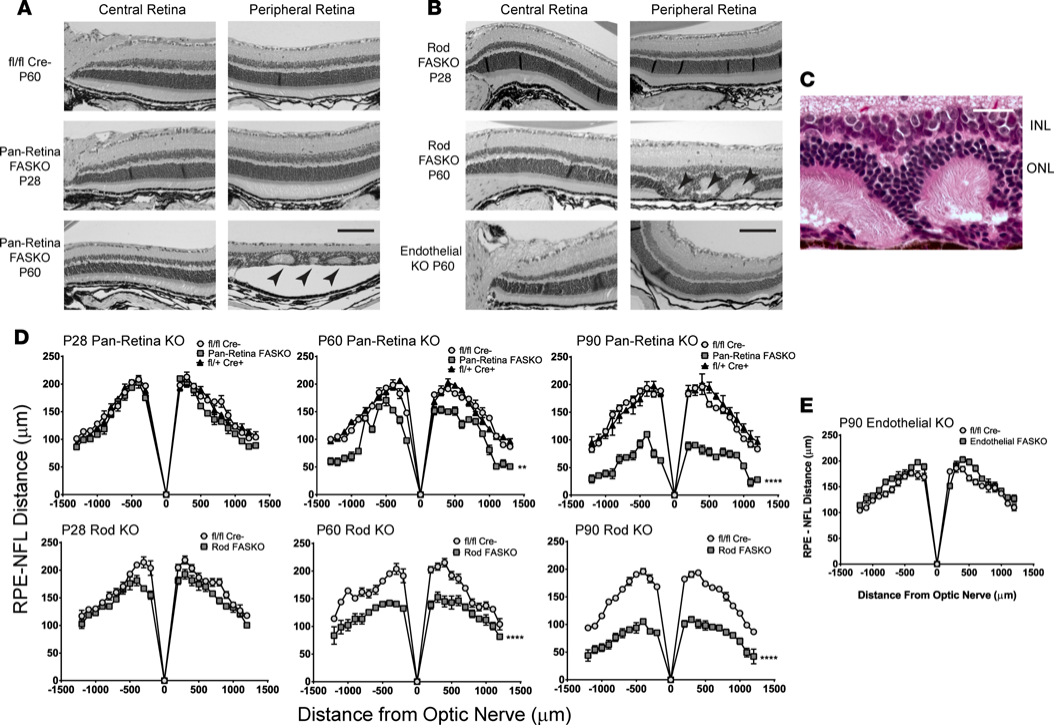
Loss of FAS activity is associated with progressive retinal degeneration. (**A**) H&E-stained paraffin cross sections of neural retinal layers, with pseudorosette formation (arrowheads) in P60 FASKO compared with P28 FASKO and to controls. (**B**) Images from rod-specific FASKO at P60 compared with P28, as well as normal images from endothelial FASKO mice. (**C**) Representative detail of outer retinal pseudorosettes seen in both rod FASKO and pan retinal FASKO, with radially arranged and apposed photoreceptor outer segments. INL, inner nuclear layer; ONL, outer nuclear layer. (**D** and **E**) Quantification of retinal thickness changes in neural and endothelial FASKO compared with littermate controls (*n* = 4 animals/group for P28, P60, and P90 Pan Retina KO, P90 Rod KO, and P90 Endothelial KO panels; *n* = 6 animals/group for P28 and P60 Rod KO panels; 2-way ANOVA). Scale bar: 100 μm (**A** and **B**) and 25 μm (**C**). **P < 0.01, ****P < 0.0001.

Disruption of REP-1, PROM1, ELOVL4, or ABCA4, each involved in lipid processing, causes early-onset retinal degeneration (6, 7, 10, 19). Like dysfunction of ELOVL4 or ABCA4, embryonic loss of retinal *Fasn* did not appear to affect eye development or function in early life. However, by P60, pan-retina FASKO eyes showed marked retinal thinning across all layers and patchy disorganization of the outer nuclear layer as compared with animals at P28 (Figure 2, A and D). Notably, pan-retina FASKO eyes develop retinal folds or pseudorosette patterns in the outer nuclear layer (Figure 2A, arrowheads, and Figure 2C), characteristic of retinal degenerative processes (20). To confirm these dramatic effects, we generated an independent FAS-deficiency model with rod-specific deletion of *Fasn* (rod FASKO) achieved with mice carrying Cre recombinase driven by the Rhodopsin promoter (21). Retinas from these mice undergo a nearly identical neurodegenerative process as pan-retinal FASKO mice, with histological appearance and retinal thickness indistinguishable from controls at P28 but dramatic retinal thinning and dysmorphology by P60 (Figure 2, B and D), including the presence of outer nuclear pseudorosettes (Figure 2B, arrowheads). Both pan-retina FASKO and rod FASKO mice displayed progressive retinal thinning in time-course experiments between P28 and P90 (Figure 2D). In contrast, an independent FAS-deficiency model with endothelial-specific deletion of *Fasn* (22) (endothelial FASKO) showed no apparent retinal thinning or dysmorphology through P90 (Figure 2E), suggesting that de novo lipogenesis in the neural retina (specifically in rod photoreceptors) — but not de novo lipogenesis in the vascular retina — is primarily responsible for maintenance of retinal structure in the adult mouse.

**Figure 3:**
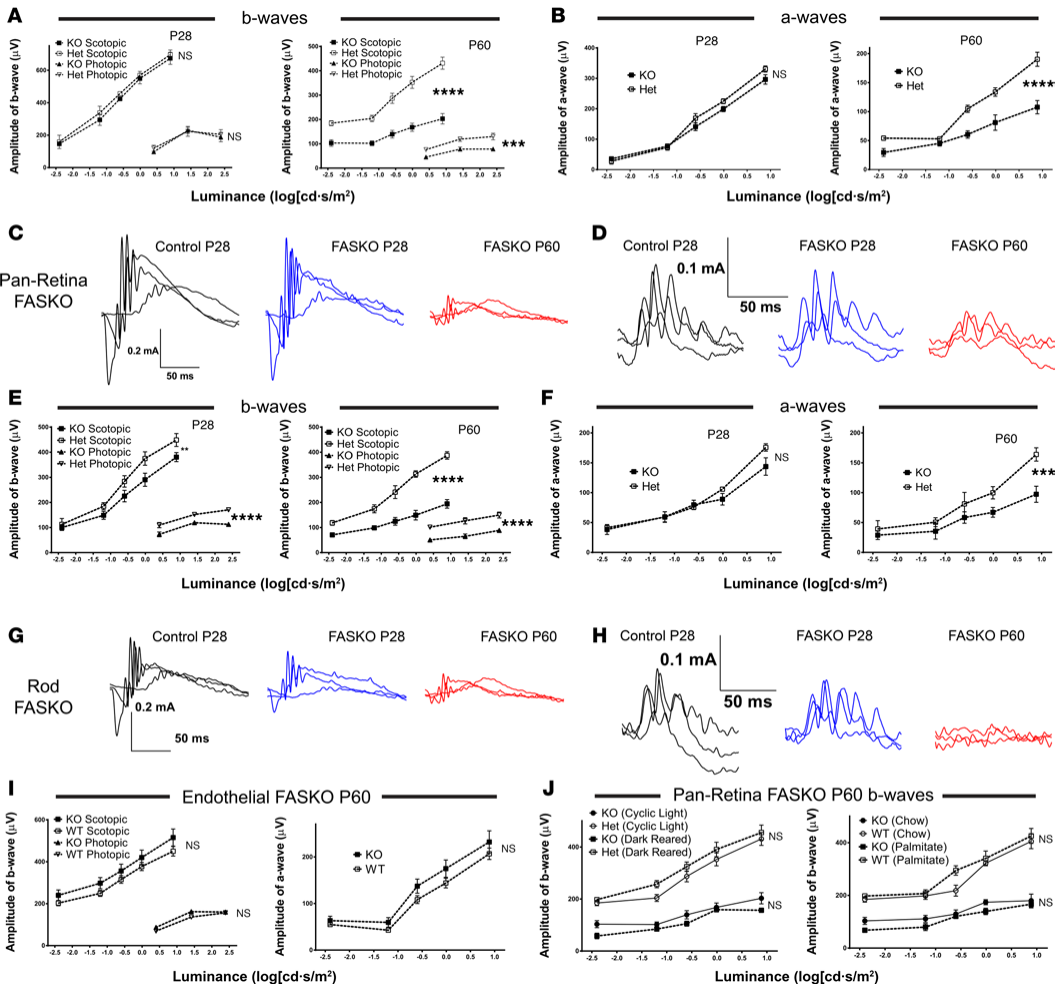
Visual function as measured by electroretinography. (**A** and **B**) b-Waves and a-waves in animals with pan-retinal deletion of FAS at P28 and P60 compared with controls under light-adapted and dark-adapted conditions (*n* = 8–10 animals/group, 2-way ANOVA). (**C** and **D**) Representative ERG tracings in scotopic (**C**) and photopic (**D**) conditions displaying marked reductions in b-wave and a-wave amplitudes across multiple stimulus luminances. (**E** and **F**) b-Waves and a-waves in animals with loss of FAS in rod photoreceptors alone (*n* = 6 animals/group, 2-way ANOVA). (**G** and **H**) Representative ERG tracings in scotopic (**G**) and photopic (**H**) conditions for mice with loss of FAS in rod photoreceptors alone. (**I**) b-Waves and a-waves in eyes with FAS deficiency in endothelial cells (*n* = 15 animals/group, 2-way ANOVA). (**J**) Effects of dark-rearing (left panel) or dietary excess of palmitate (right panel) on ERG tracings in pan-retinal FASKO mice at P60 (*n* = 8–10 animals/group; 2-way ANOVA). **P < 0.01, ***P < 0.001, ****P < 0.0001.

Retinal function in mutant mice was measured by full-field flash electroretinography. Consistent with morphologic data showing normal structure in pan-retinal FASKO mice at P28, b-waves, implicit times, and oscillatory potentials in mutant mice were comparable with littermate controls (Figure 3, A–D, and Supplemental Figure 1; supplemental material available online with this article; https://doi.org/10.1172/jci.insight.97076DS1). However, by P60, amplitudes of scotopic and photopic b-waves, as well as scotopic a-waves (Figure 3, A–D), were strikingly reduced in the KOs with maximal responses roughly half those of controls. Rod FASKO mice showed an earlier reduction in visual function and manifested progressive visual loss through P60 when b- and a-wave amplitudes were 50%–75% of controls (Figure 3, E–H). Interestingly, visual loss occurred rapidly under photopic as well as scotopic conditions, despite rod-specific deletion of FAS, consistent with bystander cone death and dysfunction reported in rod degeneration models (23, 24). Endothelial FASKO mice had visual responses comparable with controls across all ages tested under photopic and scotopic conditions (Figure 3I), suggesting that neural-compartment FAS activity is required to maintain retinal structure and function.

Because visual loss and structural degeneration of the retina only occurs after P28 in the pan-retinal FASKO model, we tested the hypothesis that light-evoked activity may induce this phenotype. We reared animals in near-complete darkness, as this can delay photoreceptor degeneration in multiple models of retinitis pigmentosa (25, 26). Unlike these mouse models, dark-reared FASKO mutants showed no difference in ERG function compared with cyclic-light exposed controls (Figure 3J, left panel). Next, we attempted phenotypic rescue of the FASKO mutant by providing palmitate — the product of the FAS biosynthetic reaction — in a diet heavily enriched for this fatty acid, beginning at P21. Pan-retina FASKO mice fed this palmitate-enriched diet (which is also enriched in cholesterol) showed ERG losses comparable with chow-fed controls (Figure 3J, right panel). FAS generates an endogenous ligand for the nuclear receptor PPARα (27), and fibrate drugs rescue the phenotype of mice with hepatic FAS deficiency (16), but feeding a chow diet supplemented with fenofibrate for 5 weeks did not rescue the visual deficits of pan-retina FASKO mice at P60 (Supplemental Figure 2).

### Deficient FAS and altered retinal membrane composition.

**Figure 4:**
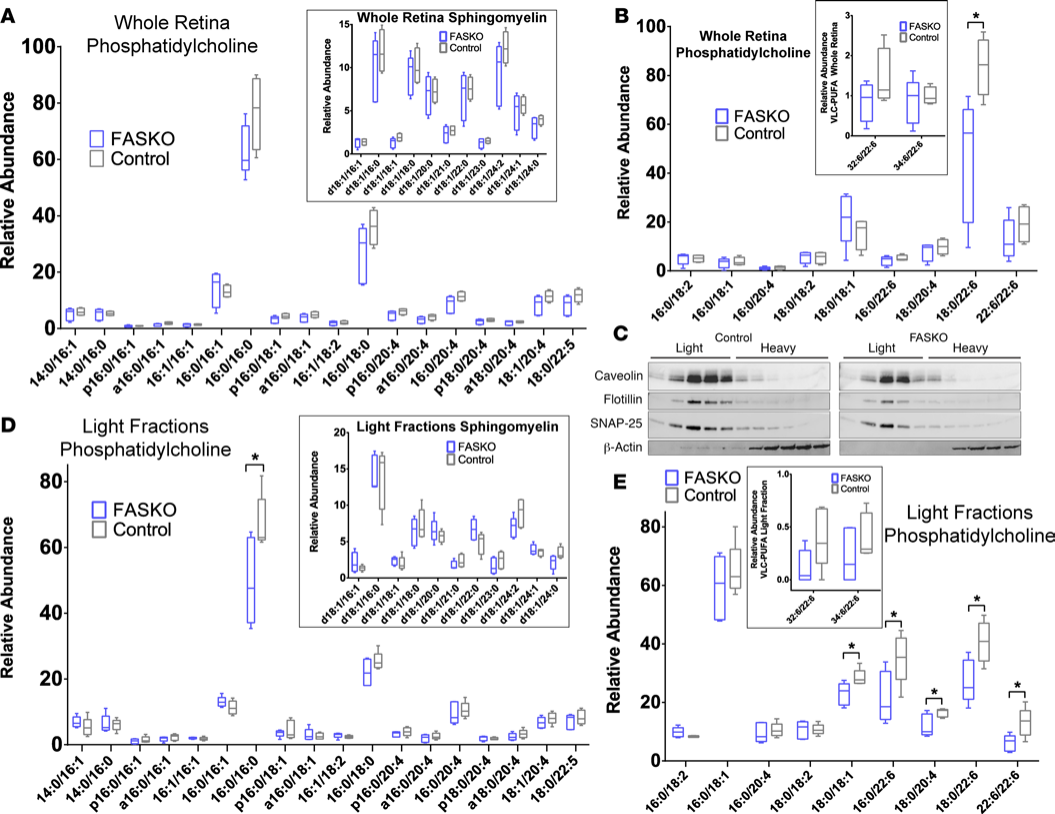
FAS deficiency and fatty acid composition of retinal membranes. (**A**) Lipid profiles in whole cell extracts for phosphatidylcholine species and sphingomyelin species (inset) in FASKO and control retina (*n* = 6 animals/group; 2-tailed *t* test). (**B**) Additional phosphatidylcholine species including DHA and other long-chain fatty acid substituents in whole cell preparations for FASKO compared with control retina, with inset showing detail for low-abundance species (*n* = 6 animals/group; 2-tailed *t* test). (**C**) Validation of microdomain isolation in FASKO and control retina. Microdomains include synaptic membranes, as indicated by positive immunoreactivity for SNAP-25. Fractions labeled as “light” were pooled and analyzed by mass spectrometry. (**D**) Lipid profiles in microdomains for phosphatidylcholine species and sphingomyelin species (inset) in FASKO and control retina (*n* = 6 animals/group, 2-tailed *t* test). (**E**) Additional phosphatidylcholine species including DHA and other long-chain fatty acid substituents in microdomains for FASKO compared with control retina, with inset showing detail for low-abundance species (*n* = 6 animals/group, 2-tailed *t* test). *P < 0.05.

Despite the profound neurodegeneration observed in FASKO retinas, mass-spectrometry of whole membranes (normalized to total protein content) showed no significant reductions in phosphatidylcholine (PC) species with substituent fatty acids containing 14–18 carbons (Figure 4A). Relative abundances of whole membrane phosphatidylethanolamine (PE), phosphatidylserine (PS), and phosphatidylglycerol (PG) species were also unchanged in FASKO retina compared with controls (Supplemental Figure 3). Sphingomyelins and ceramides were not significantly affected by FAS deficiency (Figure 4A, inset, and Supplemental Figure 3E), suggesting that the apoptosis we observed in FASKO mice was not initiated by signaling from these lipids.

Retina is heavily enriched in very long–chain fatty acids (>C26) compared with nonneural tissues. In particular, docosahexaenoic acid–containing (DHA-containing) phospholipids are abundant (28), and these species are preferentially localized to specialized membrane structures, such as synaptic membranes. In whole membranes, a major DHA-PC, 18:0/22:6-PC, was significantly reduced in FASKO retina (Figure 4B). To extend these findings, we isolated light-membrane fractions that contain lipid rafts and synaptic terminals (29). Biochemical isolations were validated by Western blotting for markers of light (caveolin, flotillin, and SNAP25) and heavy (actin) fractions (Figure 4C). No changes in abundance of any lipid species were detected in the heavy membrane fractions of FASKO mutants compared with controls (Supplemental Figure 4A). Analysis of light membrane fractions showed decreased abundance of 16:0/16:0-PC (dipalmitoyl-PC) (Figure 4D) and no significant differences in sphingomyelin species (Figure 4D, inset) or PS (Supplemental Figure 4B) with FAS deficiency. Light fraction spectra confirmed that FAS deficiency was associated with less 18:0/22:6-PC, as well as decreased abundance of the DHA-PC species 16:0/22:6-PC and 22:6/22:6-PC (Figure 4E). Two additional species, 18:0/18:1-PC and 18:0/20:4-PC (Figure 4E), were less abundant in light fractions of the pan-retina FASKO model. Alterations of DHA-PC and dipalmitoyl-PC (DPPC) content are known to contribute to early retinal neurodegeneration (19, 30).

### Deficiency of FAS disrupts retinal synapses.

**Figure 5:**
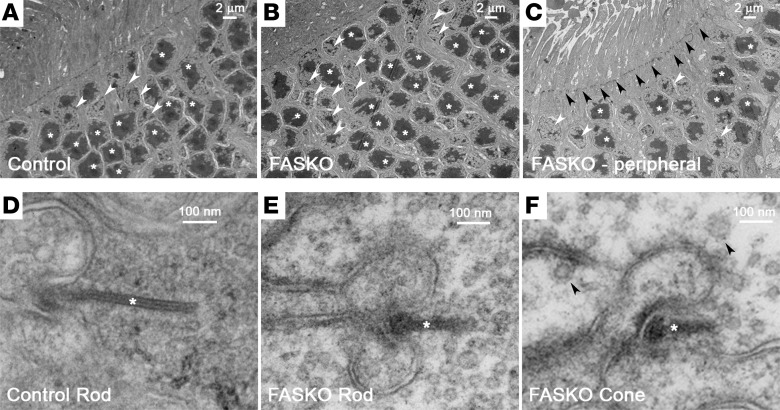
Synaptic ultrastructure in retinal FAS mutants. (**A**) Transmission electron microscopy showing control rod (asterisks) and cone (arrowhead) nuclear morphology and lamination at P28. (**B**) FASKO rod (asterisks) and cone (arrowhead) nuclear morphology and lamination at P28. (**C**) FASKO rod (asterisks) and cone (light arrowheads) nuclei at P60 around a pseudorosette with the external limiting membrane remaining intact (dark arrowheads). (**D**) Typical ribbon synapse at a rod spherule in control retina with intact ribbon strut (asterisk) and densely packed surrounding glutamatergic vesicles. (**E**) Decreased vesicular density around ribbon strut in FASKO rod spherules (asterisk) at P28. (**F**) Decreased vesicular density around ribbon strut in FASKO cone pedicles (asterisk) at P28. Clathrin-coated vesicles are indicated by arrowheads. Representative images from each panel with *n* = 2 for control tissues and *n* = 5 for FASKO tissues.

Photoreceptor outer segments, which are specialized sensory cilia, are disrupted in various forms of retinal degeneration (31). However, electron microscopy confirmed that rod photoreceptor outer segments in FAS-deficient retina were formed and intact in early life when visual function was present, similar to controls (Supplemental Figure 5, A and B, asterisks). FASKO retinas in early life also contained intact inner segment rootlet structures similar to controls (Supplemental Figure 5, C and D), normal-appearing mitochondria (Supplemental Figure 5, E and F), and normal outer segment discs (Supplemental Figure 5, G–J). Furthermore, FASKO and control rods displayed differential staining of photoreceptor discs in the proximal outer segment, characteristic of newly synthesized membranes (light vs. dark arrowheads, Supplemental Figure 5, I and J). These collective results suggest that retinal cell loss in FAS deficiency is not directly related to the integrity of cellular cilia, mitochondrial dysfunction, or loss of structural components required for photoreceptor outer segment and disc assembly. Rod and cone nuclei were arranged in regular columns in the outer layer of FASKO retina in early life in a pattern indistinguishable from age-matched littermate controls (Figure 5, A and B). At later ages in the setting of visual disruption, outer nuclear loss and disorganization were seen in the FASKO retina (Figure 5C), especially at pseudorosettes. Despite these changes, the external limiting membrane remained intact (Figure 5C, dark arrowheads).

Remarkably, ribbon synapses in FAS-depleted rods and cones showed abnormal ultrastructural features compared with controls, even early in life prior to retinal degeneration. Most striking among these changes was the reduced density of presynaptic vesicles docked to the ribbon strut and in the immediate vicinity of the synapse in FASKO retinas compared with controls (Figure 5, D–F). More clathrin-coated vesicles were present at the synaptic bouton in FASKO cone photoreceptors compared with controls (Figure 5F, arrowheads). Both features indicate early structural disorganization at the photoreceptor synaptic junction and are consistent with our ERG data showing reduced light-evoked visual function in FASKO retinas.

### Cholesterol depletion with FAS deficiency impairs retinal neurotrophic signaling.

**Figure 6:**
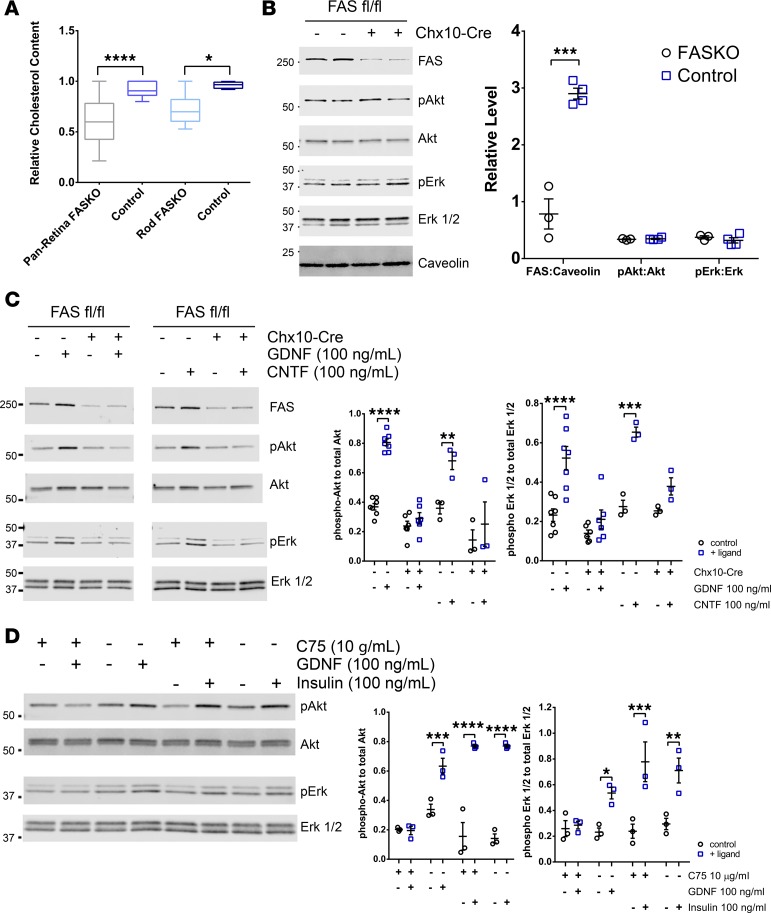
Cholesterol content and neurotrophin signaling in FASKO retina. (**A**) Retinal membrane cholesterol content in FASKO animals and animals with diabetes compared with controls (*n* = 10 animals/group; 2-tailed *t* test). (**B**) Basal Akt and ERK1/2 phosphorylation in FASKO retinal explants (*n* = 3–4 animals/group; 2-tailed *t* test). (**C**) Effects of GDNF- and CNTF-stimulated Akt and ERK1/2 phosphorylation in FASKO as compared with control retinal explants (*n* = 3–7 animals/group; 2-tailed *t* test). (**D**) Effects of chemical inhibition of FAS activity by C75 on GDNF-dependent Akt and ERK1/2 phosphorylation and on insulin signaling (*n* = 3 animals/group; 2-tailed *t* test). *P < 0.05, **P < 0.01, ***P < 0.001, ****P < 0.0001.

Synaptic formation and maintenance require cholesterol (32), and abnormal cholesterol metabolism causes severe neurological deficits, including retinal degeneration (33–35). FAS-depleted retinas had approximately 30% less total cholesterol compared with controls at P28, prior to any significant cellular loss (Figure 6A).

Since several neurotrophic signaling pathways depend on membrane microdomain integrity and lipid ordering, properties that are crucially regulated by cholesterol (36), we examined neurotrophic responsiveness in organotypic explant cultures from FASKO mice. At baseline, phosphorylation of Akt and Erk1/2, known to be engaged by canonical receptor tyrosine kinases in response to neurotrophin ligation, were not reduced in FASKO retina compared with control cultures (Figure 6B). However, phosphorylation of Akt and Erk1/2 was markedly blunted in response to glial-cell–derived neurotrophic factor (GDNF) and ciliary neurotrophic factor (CNTF) in FASKO retina, but not in controls (Figure 6C). Insulin signaling to Erk and Akt was unaffected by FAS loss in the retina (Figure 6D), suggesting selective loss of neurotrophin responsiveness in the absence of de novo lipogenesis. CNTF and GDNF signaling was also reduced in the presence of C75 (Figure 6D), a nonspecific FAS inhibitor that targets its β-ketoacyl synthase domain (37).

**Figure 7:**
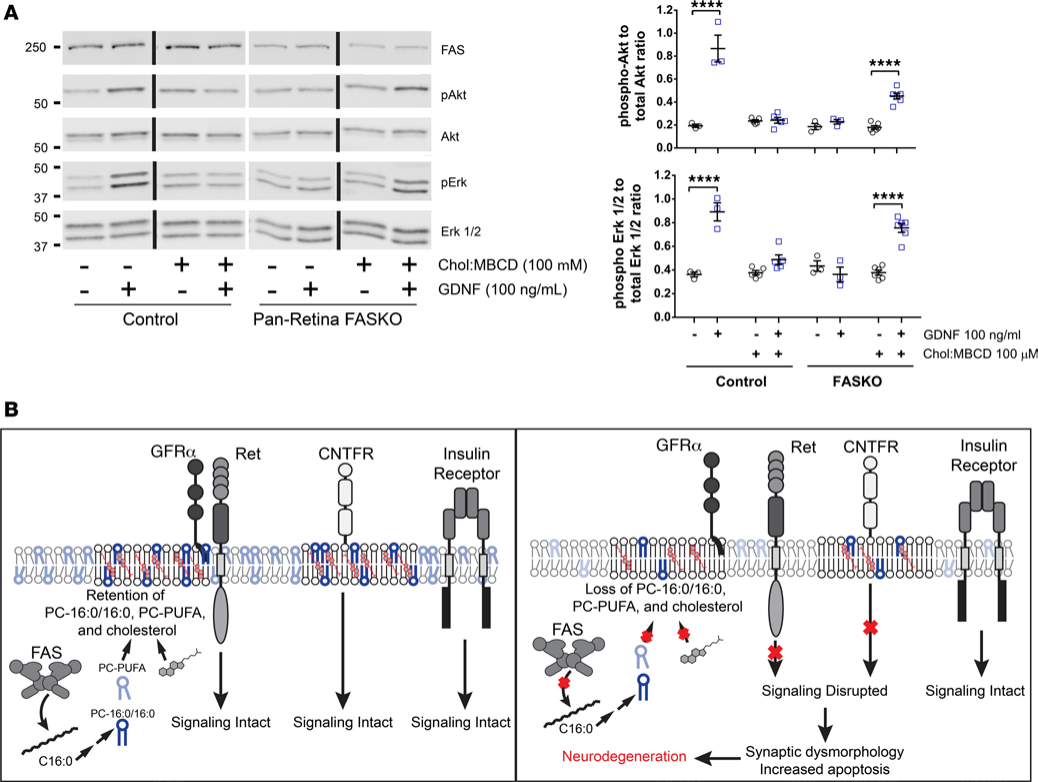
Exogenous cholesterol restores neurotrophin signaling in FASKO retina. (**A**) Effects of cyclodextrin-mediated delivery of cholesterol into retinal explants on GDNF-dependent signaling (where indicated by dark bars, lanes were run on the same gel but were noncontiguous) (*n* = 3 animals/group; 1-way ANOVA). ****P < 0.0001. (**B**) Model for proposed role of FAS in maintaining photoreceptor survival and synaptic integrity by facilitating cholesterol and fatty acid retention in plasma membrane microdomains where neurotrophin receptor signals are initiated. GRFα, GDNF family receptor α.

To determine whether FAS-dependent plasma membrane sterol loss caused decreases in neurotrophin responsiveness, we replenished cholesterol in the explants using methyl-β cyclodextrin (MBCD) delivery (38). To ensure adequate cholesterol repletion, we delivered cholesterol complexed to MBCD both by vascular infusion prior to retinal extraction and into the bathing media of the explant itself. Indeed, these combined measures restored GDNF-mediated signaling in FAS-deficient retinal explants (Figure 7A). Notably, cholesterol delivery to control retinas blunted GDNF signaling, suggesting that cholesterol content is tightly regulated in retinal membranes to maintain appropriate neurotrophic support.

## Discussion

Retinas lacking fatty acid synthase develop normally but — independent of light exposure, high-fat feeding, or fenofibrate treatment — subsequently undergo rapidly progressive neurodegeneration characterized by global loss of retinal neurons and resembling retinitis pigmentosa. These retinas contain aberrant synapses with reconfiguration of membrane fatty acid composition and marked reductions in total cholesterol content (Figure 7B), leading to multiple adverse effects on neural physiology, including decreased neurotrophic signaling that is partially rescued by delivery of exogenous cholesterol.

In contrast to loss of lysophosphatidic acid acyltransferase, a terminal enzyme in retinal DHA-phospholipid generation (39), FAS loss in the retina did not prevent normal assembly of outer segment discs in cones or rods (Supplemental Figure 5, G–J), suggesting that FASKO retinas undergo neurodegeneration through distinct molecular mechanisms. Two lipid species linked to retinal neurodegeneration, DPPC, and n-3 fatty acid–containing PCs were reduced in light membrane fractions of the FASKO retina. Linkage analysis in retinal degeneration 11 (rd11) mice that undergo rapid photoreceptor loss identified a mutation in the coding region of the *Lpcat1* (30), which encodes for an acyl transferase that synthesizes DPPC from C16-containing precursors. Dietary supplementation with DPPC does not rescue the rd11 phenotype (30). Reductions in content or biosynthesis of the n-3 fatty acid DHA are associated with retinal degeneration in humans and mice (40, 41). Increased DHA dietary intake elevates circulating levels of DHA, but this intervention is insufficient to protect vision in humans or mice with lipid-related retinopathies. Moreover, neural retina DHA content does not correlate with content in adipose depots, suggesting that a recycling pathway intrinsic to the eye may be important for n-3 fatty acid effects on vision (42). In the current work, exogenous palmitate supplementation did not prevent vision loss in FASKO mice, consistent with the existence of de novo lipogenesis–dependent pathways intrinsic to the eye that are important for vision.

Cholesterol content was decreased in FASKO retina, and dysregulation of cholesterol homeostasis has profound effects on retinal structure and function. In Smith-Lemli-Opitz syndrome (SLOS), defective 3 β-hydroxysterol-δ7-reductase activity decreases cholesterol and increases 7-dehydroxy-cholesterol, changes associated with retinal degeneration and marked rod photoreceptor dysfunction (33, 43, 44). In a rodent model of SLOS, there is rapid retinal degeneration and loss of both rod and cone photoreceptor function that are partially restored by the feeding of a high-cholesterol diet (45). Similar to our findings in FASKO mice, DHA-containing phospholipids are inefficiently retained at the cell membrane in the SLOS rat (46). It is unclear whether cell loss in SLOS is due to cholesterol deficiency or accumulation of precursor molecules. One possibility is that both SLOS and FASKO eyes are characterized by abnormal interactions between cholesterol and phospholipids that disrupt the integrity of membrane microdomains required for signaling, synaptic transmission, and survival. In SLOS retina, a primary defect in sterol metabolism alters recruitment of membrane phospholipids essential for lipid raft integrity. In FASKO retina, a primary defect in lipogenesis depletes critical membrane phospholipids essential for retaining cholesterol required to initiate signaling from lipid rafts. The retinal FAS effect is analogous to signaling in macrophages, where FAS is required for maintaining lipid raft phospholipid composition, retaining membrane cholesterol, and promoting receptor-mediated intracellular signaling (47).

Neuronal death in FASKO mice contributes to a body of evidence supporting the notion that retinal lipid metabolism, independent of exogenous lipid delivery, is critical for preventing retinal degeneration. The cause of cell death is unclear in many retinal degenerative diseases, despite compelling evidence of responsible genetic abnormalities. In Stargardt disease 3, decreased Elovl4 activity is associated with cell death through unknown mechanisms. It is plausible that abnormal cholesterol content in this and other forms of inherited retinopathy may contribute to cell death by disrupting neurotrophic signaling. Mutant mice lacking GDNF-family ligands or the receptor for these ligands, Ret, manifest retinal degeneration in early adulthood (48), a process resembling defective GDNF signaling and retinal degeneration in FASKO retina. Supplementation with neurotrophins in patients with retinitis pigmentosa fails to prevent visual loss (49, 50), but these results could be due to insufficient cholesterol-dependent engagement of signaling machinery. If so, targeted cholesterol repletion could increase the efficacy of neurotrophin supplementation to treat retinal degeneration. Such an approach would likely require repletion of cholesterol within a narrow range. While cholesterol delivery restored neurotrophic signaling in FAS deficiency, it decreased signaling in control retinal explants.

Mutations in *FASN* have not been described that cause human retinal degeneration, perhaps because most of these mutations would be predicted to be lethal (51). However, FASKO retina mirrors several established rodent models of retinal degeneration, as well as degenerative retinopathies in humans. In particular, FASKO retinas are deficient in fatty acid intermediates (DHA-linked PC and DPPC) that are also deficient in mice with retinal neurodegeneration due to defects in *Elov4* and *Lpcat1*. FASKO retinas form outer retinal pseudorosettes like those seen in retinitis pigmentosa. FASKO retinas also lack appropriate levels of membrane cholesterol, similar to SLOS retinas. Moreover, dietary fatty acid supplementation does not rescue the degenerative phenotype of the FASKO retina, just as this intervention does not prevent blindness in human retinopathies. But notably, steady-state levels of palmitate were normal in the FASKO retina. Together, these findings strongly suggest that photoreceptors can distinguish locally synthesized pools of palmitate from those that are obtained from extracellular sources and that these de novo pools are essential for maintaining retinal integrity over early adulthood. Some of this newly synthesized palmitate is required for retaining membrane cholesterol and maintaining responsiveness to appropriate trophic signals, as we have shown. However, locally generated palmitate may also be required for other aspects of photoreceptor physiology that could be relevant to related retinal degenerative conditions. Therefore, retinal de novo lipogenesis is likely to be important for protection from several forms of inherited retinopathy.

## Methods

### Animals.

C57BL/6J mice were free of rd1 or rd8 mutations. Animals were fed Purina 4043 (13% kcal from fat, 62% kcal from carbohydrate, 25% kcal from protein) or Harlan Teklad TD 88137 (42% kcal from fat, 43% kcal from carbohydrate, 15% kcal from protein).

### Antibodies and PCR primers.

For Western blotting, we used polyclonal rabbit IgG against FAS (catalog ab22759, AbCam), polyclonal rabbit IgG against Akt phosphorylated at S473 (catalog 4060, Cell Signaling Technology), mouse monoclonal IgG1 against pan-Akt (catalog 2920, Cell Signaling Technology), mouse monoclonal IgG1 against Erk1/2 phosphorylated at T202/Y204 (catalog 9106, Cell Signaling Technology), rabbit polyclonal IgG against Erk1/2 (catalog 9102, Cell Signaling Technology), rabbit polyclonal IgG against SNAP-25 (catalog 3926, Cell Signaling Technology), rabbit polyclonal IgG against caveolin-1 (catalog sc-894, Santa Cruz Biotechnology Inc.), mouse monoclonal IgG1 against flotillin-1 (catalog 610820, BD Biosciences), and rabbit polyclonal IgG against actin (catalog A2066, MilliporeSigma). For immunostaining, anti-FAS was used at 1:250.

PCR primers were: *Fasn* sense 5′ - GTCGTCTGCCTCCAGAGC - 3′; *Fasn* antisense 5′ - GTTGGCCCAGAACTCCTGTA-3′; *Lpcat1* sense 5′ - CACGAGCTGCGACTGAGC - 3′; *Lpcat1* antisense 5′ - GAAGCCAGGAGTGCAAAGG - 3′; *Srebf1* sense 5′ - GGCTCTGGAACAGACACTGG - 3′; *Srebf1* antisense 5′ - TGGTTGTTGATGAGCTGGAG - 3′; *Elovl4* sense 5′ - ACCGTGGAGTTCTATCGCTG - 3′, *Elovl4* antisense 5′ - GCTTATGCTTATCGTTGGC - 3′; *Gapdh* sense 5′ - TGCACCCCAACTGCTTAGC - 3′; *Gapdh* antisense 5′ - GGCATGGACTGTGGTCATGAG - 3′; *Rpl32* sense 5′ - GGCTTTTCGGTTCTTAGAGGA - 3′; and *Rpl32* antisense 5′ - TTCCTGGTCCACAATGTCAA - 3′. Gene expression data were normalized to the mean of 2 internal controls, *Gapdh* and *Rpl32*.

### FAS enzyme activity.

We determined FAS enzyme activity by 2 methods (16, 52). For the NADPH depletion assay, 2 isolated retinas from each animal were combined and homogenized in 100 μl of 0.1 M potassium phosphate buffer (pH 7.0) with 8% sucrose and 1 mM EDTA (catalog E-5134, MilliporeSigma) at 4°C. Homogenates were cleared by centrifugation at 3,000 *g* for 5 minutes. Total protein was quantified by Bradford Assay. Normalized sample (90 μl) containing 10 μg of protein was added to 35 μl of an assay buffer containing 0.1 M potassium phosphate (pH 7.0), 1 mM EDTA, 1 mM DTT (catalog D-0632, MilliporeSigma), and 0.4 mg/ml NADPH (catalog N-1630, MilliporeSigma). The rate of NADPH oxidation was monitored at 340 nm, before and after the addition of 10 μl of 0.85 mg/ml malonyl-CoA (catalog M-4263, MilliporeSigma). Substrate-dependent enzyme activity was determined by calculating the difference between these 2 measures.

For the ^14^C-Malonyl-CoA assay, 2 isolated retinas per animal were homogenized in lysis buffer and centrifuged as above. Supernatant (100 μl) was added to a buffer containing 0.1 M potassium phosphate buffer (pH 7.0), 1 mM acetyl-CoA (catalog A-2056, MilliporeSigma), 1 mM cold malonyl-CoA, 0.1 μCi ^14^C-malonyl-CoA (catalog NEC612005UC, Perkin Elmer), 1 mM dithiothreitol, 1 mM EDTA, and 0.5 mM freshly prepared NADPH. This mixture was incubated in a 37°C agitating bath for 15 minutes, and reactions were then terminated using 7.5 μl of 60% perchloric acid (catalog 244252, MilliporeSigma) with immediate vortexing. The resulting cloudy mixture was treated with 0.25 ml of absolute ethanol. Then, 0.75 ml petroleum ether (catalog 32299, MilliporeSigma) was added, and the mixture was agitated and centrifuged to generate an organic phase containing long-chain fatty acids. Three such extractions with petroleum ether were performed, and the pooled organic phase was added to a scintillation counter to quantitate the radiolabel.

### NADPH measurement.

Frozen tissue samples were rapidly extracted in ice-cold potassium hydroxide and then neutralized in monopotassium phosphate. NADPH concentrations were determined using an HPLC system (Prominence; Shimadzu Scientific Instruments) with a Supelco LC-18-T column (catalog 58970-U; MilliporeSigma). The HPLC conditions were as described previously (53, 54). NADPH concentrations were normalized to tissue weights.

### Retinal morphologic assays.

Eyes from male and female mice were fixed in 4% paraformaldehyde (catalog O4042-500, Thermo Fisher Scientific) at 4°C for 48 hours, paraffin-embedded, sectioned into 4-μm slices, and stained with H&E. For TUNEL staining, paraffin-embedded sections were dewaxed and subjected to antigen retrieval. Then, an in situ anti-digoxigenin antibody labeling kit (ApopTag Red, catalog S7165, MilliporeSigma) was used. The number of positive cells per high-power field (0.25 mm^2^) was quantitated using ImageJ software (NIH).

### Electroretinography.

A UTAS BigShot System (LKC Technologies Inc.) was utilized as described (55). Mice (>5 for each group) were dark-adapted overnight. Under red-light illumination, animals were anesthetized with ketamine (80 mg/kg) and xylazine (15 mg/kg). Pupils were dilated with 1% atropine sulfate; body temperature was maintained at 37°C with a heating pad. Contact lens electrodes were placed bilaterally with appropriate reference and ground electrodes.

The stimulus consisted of a full-field white-light flash (10 μs) either in darkness or in the presence of dim (30.0 cd/m^2^) background illumination after a 10-minute adaptation time. The response was recorded over 231 ms plus 25 ms of pretrial baseline. Between 5 and 10 repeated trials were averaged for each luminance, with 10 repeats used for the dimmest flashes and 5 for the brightest. Raw data were processed using a MATLAB program (MathWorks). The amplitude of the a-wave was measured from the average pretrial baseline to the most negative point of the average trace, and the b-wave amplitude was measured from that point to the highest positive point. The log luminance of the stimulus (log [cd·s/m^2^]) was calculated based on manufacturer’s calibrations.

### Lipid analyses by electrospray ionization mass spectrometry.

Two retinas from each animal were freshly isolated, pooled into 500 μl of 40% methanol, and homogenized in a glass tube with a Dounce homogenizer. An aliquot of each homogenate (50 μl) was reserved and diluted in deionized water to 10% methanol for total protein measurement using Bradford reagent. Using the remaining homogenate, an appropriate amount of each internal standard was added before Folch extraction (56). After extraction, the organic layer was collected, dried under nitrogen, and reconstituted in 200 μl chloroform/methanol (1:1) with 0.1% NH_4_OH. A 10-μl aliquot was loop injected into a Thermo Vantage triple quadrupole mass spectrometer using an Accela autosampler with 1250 HPLC pump, which delivered a constant flow of 40 μl/min of methanol with 0.1% NH_4_OH. Analyses of PC and sphingomyelin species were carried out in the positive ion mode using a precursor scan of 184 with a collision energy of 33 eV to detect the molecular species as the [M + H]^+^ ions. Ceramides were detected as the [M – H]^–^ ions via a neutral loss scan of 256 using a collision energy of 32 eV. PE species were detected as the [M – H]^–^ ions in the negative ion mode using a precursor ion scan of 196 with a collision energy of 50 eV or similarly in positive ion mode as [M + H]^–^ ions with a precursor scan of 141. A precursor ion scan of 153 with a collision energy of 32 eV, and a neutral loss scan of 87 under a collision energy of 25 eV, were used for measurement of PG and PS species as the [M – H]^–^ ions in the lipid extract, respectively. Quantitation of each individual PC, PE, PS, and PG species was compared with the 14:0/14:0-PC, 14:0/14:0-PE, 14:0/14:0-PS, and 14:0/14:0-PG internal standards, respectively, and results were normalized to the total protein content of the input.

### Free fatty acid detection by mass spectrometry.

N-(4-aminomethylphenyl)pyridinium (AMPP) derivative was made with a kit (AMP+ Mass Spectrometry Kit, catalog 710000, Cayman Chemical), according to the manufacturer’s instructions. Dissected retinas were combined with a deuterium-labeled internal standard (Tridecanoic-d_25_ Acid, catalog D-4002, CDN Isotopes), subjected to Folch extraction, and dried as described above. The dried sample was resuspended in 50 μl ice-cold acetonitrile/dimethylformamide (DMF) (4:1, v/v). To this solution, 50 μl of ice-cold 1M EDCI (3-[(dimethylamino)propyl]ethyl carbodiimide hydrochloride) in water was added, and the mixture was vortexed. Then, 25 μl of 5 mM N-hydroxybenzotriazole (HOBt) and 75 μl of 15 mM AMPP in distilled acetonitrile were added, mixed, and heated at 60°C for 30 minutes. After cooling to room temperature, 500 μl water and 500 μl n-butanol were added. The final solution was vortexed for 1 minute and centrifuged at 1,200 *g* for 3 minutes, and the organic layer was transferred to another vial. The FA-AMPP derivative was analyzed using a Bruker Daltonics UltrafleXtreme time of flight (TOF)/TOF spectrometer equipped with a smartbeam-II laser, which is operated at a repetition rate of 2 kHz for MS acquisition. α-Cyano-4-hydroxycinnamic acid (CHCA) matrix was dissolved in 1:1 water/acetonitrile containing 0.1% trifluoroacetic acid at a concentration of 10 mg/ml and premixed with the derivatives (1:1, v/v) in a vial. A 1.0-μl aliquot of this mixture was deposited onto a MALDI target plate for analysis. Fatty acid peaks were normalized to protein concentrations and to the internal standard.

### Isolation of membrane microdomains.

To isolate light membrane fractions, dissected retinas (2 from each animal, combined) were lysed in 500 μl of high pH buffer containing 500 mM sodium carbonate (pH 11.0) and protease inhibitors (Roche Diagnostics), incubated on ice for 30 minutes, and homogenized by sonication at 25% amplitude for 20 seconds with a 50% duty cycle. Homogenates were adjusted to 800 μl of 45% sucrose and placed under sucrose layers of 5% (450 μl) and 35% (1 ml). After centrifugation at 184,000 *g* in a TLS-55 rotor (Beckman) at 4°C for 16 hours, 200-μl fractions were sequentially collected from top to bottom. Fractions were analyzed by SDS-PAGE and immunoblotting. Fractions 1–5 were combined as the light membrane component, and fractions 6–11 were considered the heavy membrane component. Combined fractions were subjected to Folch total lipid extraction and analyzed by electrospray ionization mass spectrometry as described above.

### Transmission electron microscopy.

Mice were deeply anesthetized with ketamine (80 mg/kg) and xylazine (15 mg/kg) and, after thoracotomy, perfused through the left ventricle with 15 ml mammalian Ringer’s buffer over 15 minutes, followed by 15 ml freshly prepared 2.5% glutaraldehyde in 0.1 M cacodylate buffer (pH 7.35) for another 15 minutes. After enucleation and removal of the cornea and lens, eye cups were then immersion-fixed overnight in 2.5% glutaraldehyde buffer at 4°C. They were then postfixed in 1% osmium tetroxide for 1 hour and stained with 1% uranyl acetate in 0.1 M acetate buffer (pH 7.4) for 1 hour. Tissues were dehydrated in a graded series of ethanol and then prepared for embedding with increasing concentrations of propylene oxide (Electron Microscopy Sciences). Retinal cups were then embedded in Araldite 6005/EMbed 812 resin (Electron Microscopy Sciences), cut through the optic nerve at 0.5–1 mm depth and stained with toluidine blue and examined by light microscopy. Ultra-thin sections were prepared and poststained with uranyl acetate and lead citrate and were viewed and photographed on a JEOL JEM-1400 transmission electron microscope.

### Cholesterol measurements.

Freshly isolated retinas (2 per animal) were pooled, weighed, and homogenized in a 1.5 ml mixture of chloroform and methanol (2:1 v/v). The homogenate was centrifuged at 13,400 *g* for 10 minutes at 4°C. The upper organic phase (100 μl) was evaporated in a 1.5-ml tube at room temperature for 30 minutes. Once dried, 100 μl of the cholesterol detection reagent (Infinity Total Cholesterol reagent, catalog TR13421, Thermo Fisher Scientific) was added to the tube and allowed to incubate at room temperature for 30 minutes. The mixture was transferred to a 96-well dish, and absorbance at 490 nm was determined. Absolute quantification was achieved by comparing results with a standard curve, and all measurements were normalized to input retinal mass. Results are reported relative to each biologic control.

### Retinal responsivity to extracellular ligands.

Human insulin was from MilliporeSigma (catalog I-5523) and rat CNTF was from R&D Systems (catalog 557-NT-010). Rat GDNF was a gift of Yo Sasaki and Jeffrey Milbrandt (Washington University).

Animals were deeply anesthetized and sacrificed by cervical dislocation. Retinas were immediately isolated into DMEM supplemented with 20 mM glucose and penicillin plus streptomycin. Explants were incubated at 37°C in a tissue culture incubator (Forma Scientific) with 5% CO_2_ for 2 hours. Ligands were added at the indicated concentrations directly into the dish and allowed to incubate for 40 minutes at 37°C. Explants were then transferred into a centrifuge tube, and excess media was aspirated. The tissue was washed with 1 ml cold PBS and subsequently homogenized in 150 μl of RIPA buffer (10 mM Tris, 1% Nonidet P-40, 1 mM EDTA, 10% glycerol, 0.1% sodium dodecyl sulfate supplemented with protease inhibitors from Roche Diagnostics). After 30 minutes on ice, samples were centrifuged at 10,000 *g* for 10 minutes at 4°C. Supernatants were collected and analyzed for total protein content using BCA chemistry. Normalized samples were boiled in 1× Laemmli sample buffer for 5 minutes and analyzed by SDS-PAGE and subsequent immunoblotting.

### Exogenous cholesterol rescue of neurotrophin responses.

Cholesterol (catalog C3045, MilliporeSigma) was complexed to methyl-β-cyclodextrin (catalog C4555, MilliporeSigma) at a ratio of 1:10 (with 0.25 mM cyclodextrin) and diluted to desired concentrations using serum-free medium. Cholesterol was dried under nitrogen gas and sonicated into either normal saline (for perfusion) or DMEM (for treatment of explants) with cyclodextrin until the precipitate disappeared. The solution was agitated overnight at 37°C and then passed through a 0.2-μm filter. After inducing deep anesthesia with ketamine (80 mg/kg) and xylazine (15 mg/kg), followed by thoracotomy, animals were perfused through the left ventricle with either 20 ml of 100 μM cholesterol complexed to cyclodextrin or 20 ml of normal saline over 20 minutes. Retinas were immediately dissected into serum-free DMEM alone or supplemented with 100 μM cholesterol with cyclodextrin for an additional 20 minutes. Following these pretreatments, explants were exposed to GDNF (100 ng/ml) or vehicle (0.1% BSA) for 40 minutes and isolated for immunoblotting analyses.

### Statistics.

In line graphs, data are expressed as mean ± SEM. In box-and-whisker plots, data are expressed as median, with the box showing the limits of the interquartile range, and whiskers representing maxima and minima. For experiments with 2 groups in the independent variable, analyses were performed using 2-tailed *t* test without posthoc correction. For experiments with more than 2 groups in the independent variable, 1-way ANOVA with Bonferroni correction was used when only 1 dependent variable was present, and 2-way ANOVA with Bonferroni post-tests was used when 2 or more dependent variables were present. All calculations were performed using GraphPad Prism 6.0 software. In all experiments, *P < 0.05, **P < 0.01, ***P < 0.001, ****P < 0.0001.

### Study approval.

Protocols followed the Association for Research in Vision and Ophthalmology Statement for the Use of Animals and were approved by Washington University.

## Author contributions

RR, JY, and FFH designed studies, conducted experiments, acquired data, analyzed data, and wrote the manuscript. SZ, XW, TD, SA, JE, VS, GL, and SC conducted experiments and acquired data. CFS designed studies, analyzed data, and wrote the manuscript.

## Supplementary Material

Supplemental data

## Version 1 01/11/2018

Electronic publication
