# Supplementary material for: Retinal de novo lipogenesis coordinates neurotrophic signaling to maintain vision
Source: JCI Insight. 2018 Jan 11;3(1):e97076. doi: 10.1172/jci.insight.97076 (PMC5821215; doi:10.1172/jci.insight.97076)
Supplement: Supplemental data [file jciinsight-3-97076-s001.pdf]

Figure S1

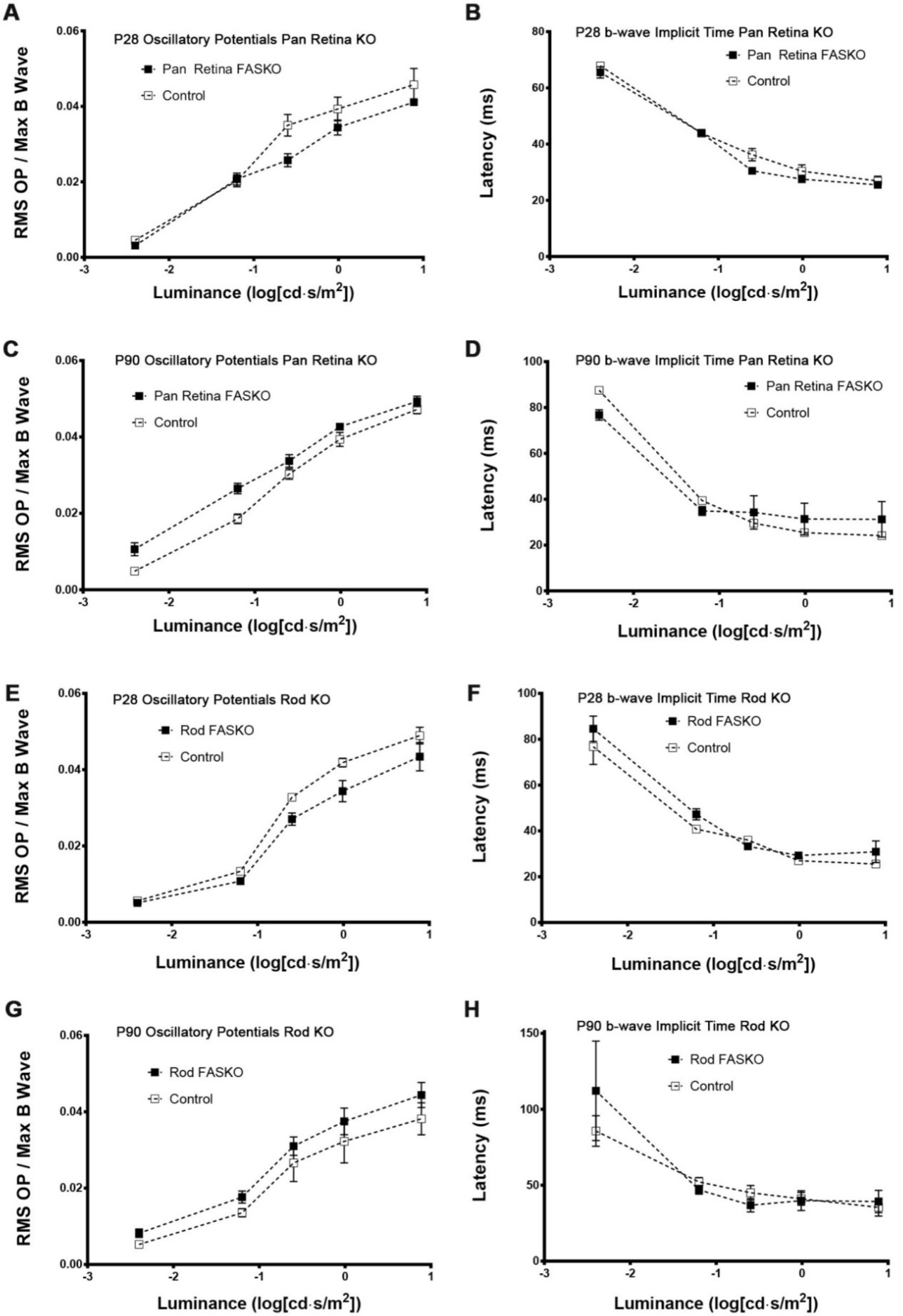

Supplemental Figure 1. Additional electroretinographic properties of FASKO animals. (**A**, **C**) Scotopic oscillatory potentials (OP) in P28 (A) and P90 (C) animals with panretinal FAS deletion, represented as the root mean square (RMS) of their amplitudes normalized to the maximal b-wave amplitude (n=8-10 animals/group, two-way ANOVA). (**B**, **D**) Implicit times of the scotopic b-waves in pan-retina FAS KO animals at P28 (B) and P90 (D) (n=8-10 animals/group, two-way ANOVA). (**E**, **G**) Scotopic OPs in rod FASKO at P28 (E) and P90 (G) (n=6 animals/group, two-way ANOVA). (**F**, **H**) Implicit times of scotopic b-waves in rod FASKO at P28 (F) and P90 (H) (n=6 animals/group, two-way ANOVA). $P<0.05$ was considered significant.

Figure S2

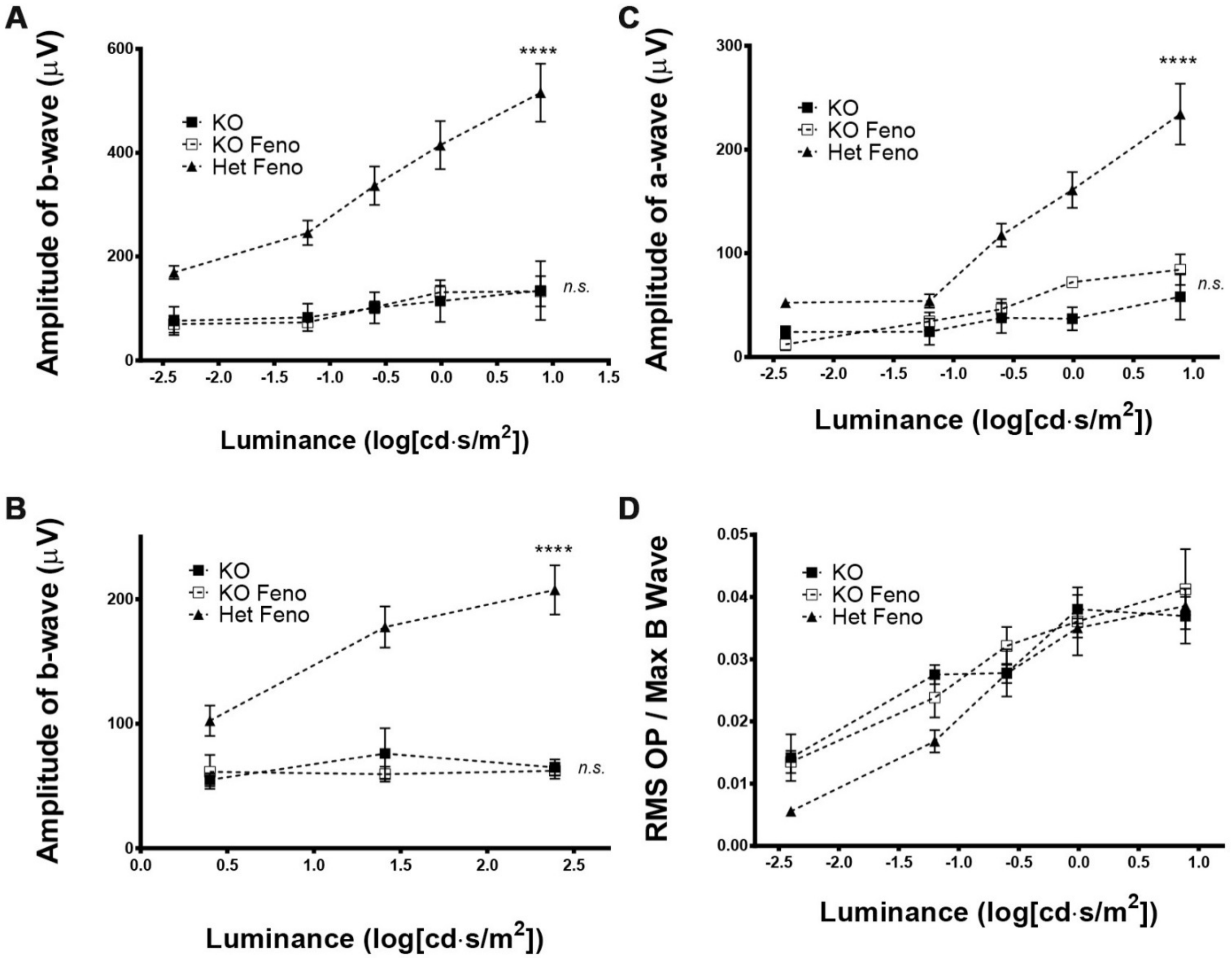

Supplemental Figure 2. Electroretinography in FASKO after chronic fenofibrate feeding. (**A**, **C**) dark-adapted b-waves and a-waves in P60 pan-retina FASKO (KO Feno) or heterozygous controls (Het Feno) after 5 weeks of feeding with 0.2% (w/w) fenofibrate-supplemented diet or standard chow (KO). (**B, D**) Photopic b-waves (B) and scotopic OPs (D) after fenofibrate feeding. For all experiments, n=4 animals in KO group, 10 animals in KO Feno group, and 14 animals in Het Feno group. **** indicates P<0.0001 (Two-way ANOVA).

Figure S3

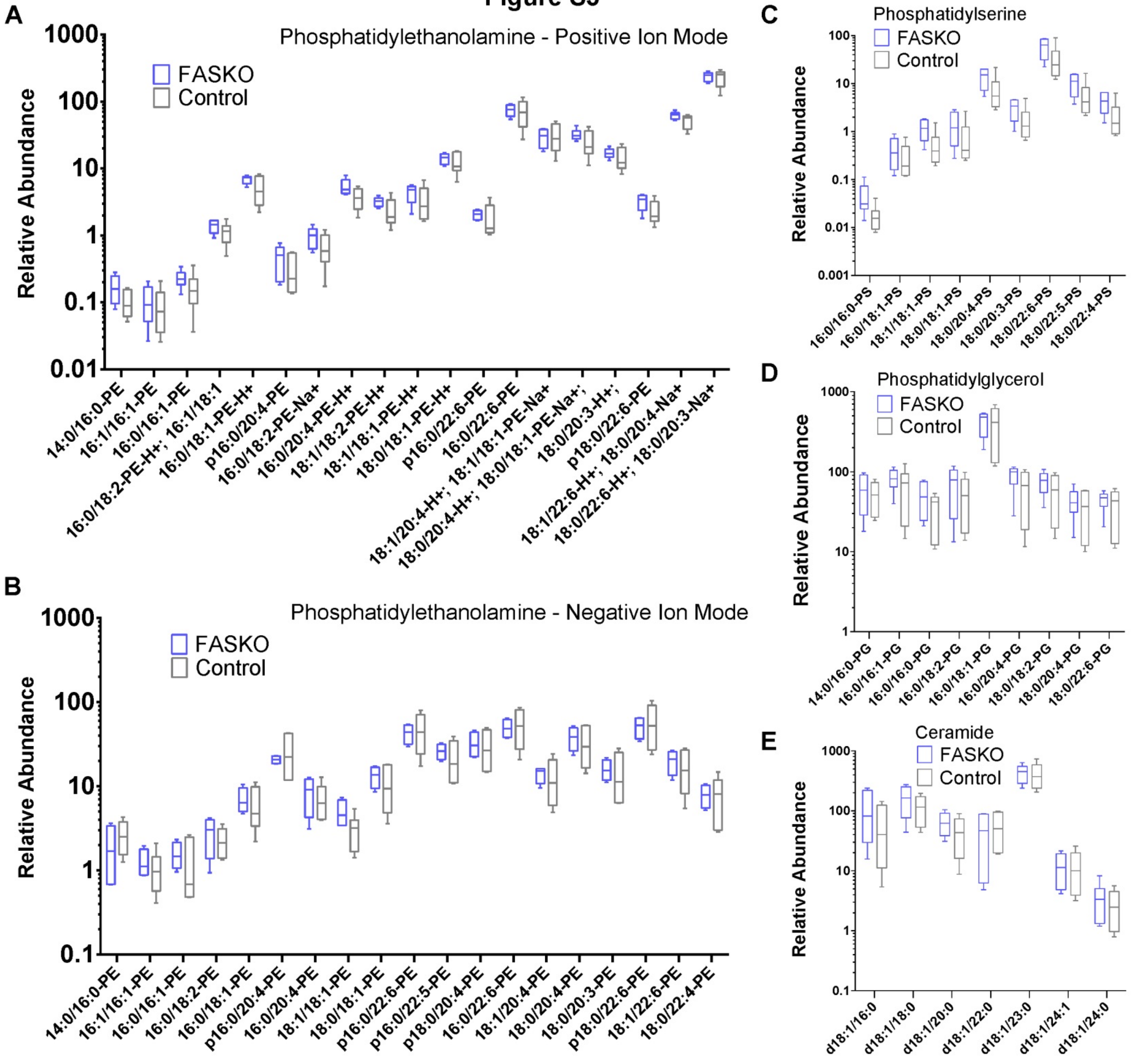

Supplemental Figure 3. Phospholipid profiling of whole retinal FASKO membranes at P28. (**A**, **B**) Quantitation of phosphatidylethanolamines detected by positive (A) and negative (B) ion mode LC-ESI-MS. (**C**, **D**, **E**) Whole retinal lipid profiles for phosphatidylserines (C), phosphatidylglycerols (D) and ceramides (E). For all experiments, n=6 animals/group, analyses by t-test. P<0.05 was considered significant.

Figure S4

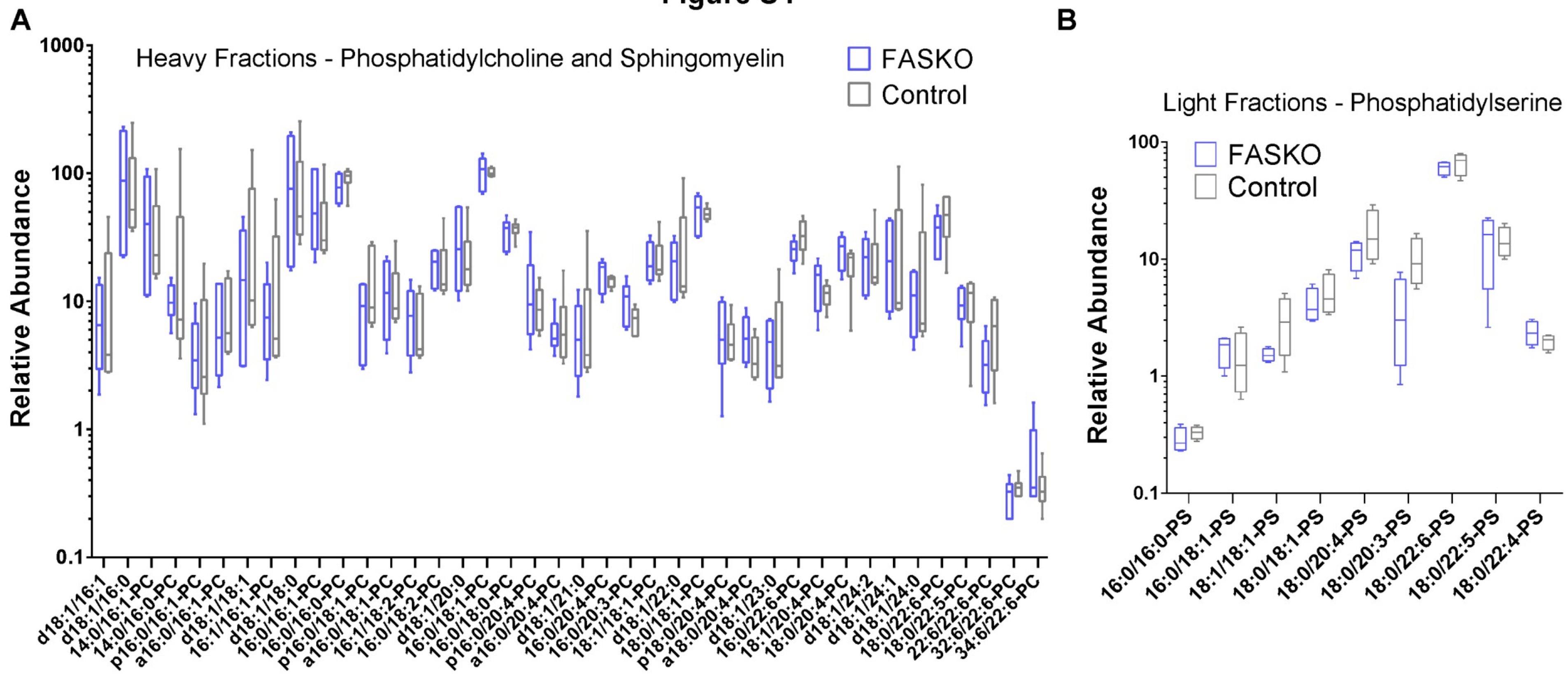

Supplemental Figure 4. Additional lipid analysis in P28 FASKO. (**A**) Phosphatidylcholine and sphingomyelin species detected in heavy membrane fractions. (**B**) Phosphatidylserine quantification from light membrane fractions. For all experiments, n=6 animals/group, analyses by t-test, *P*<0.05 was considered significant.

Figure S5

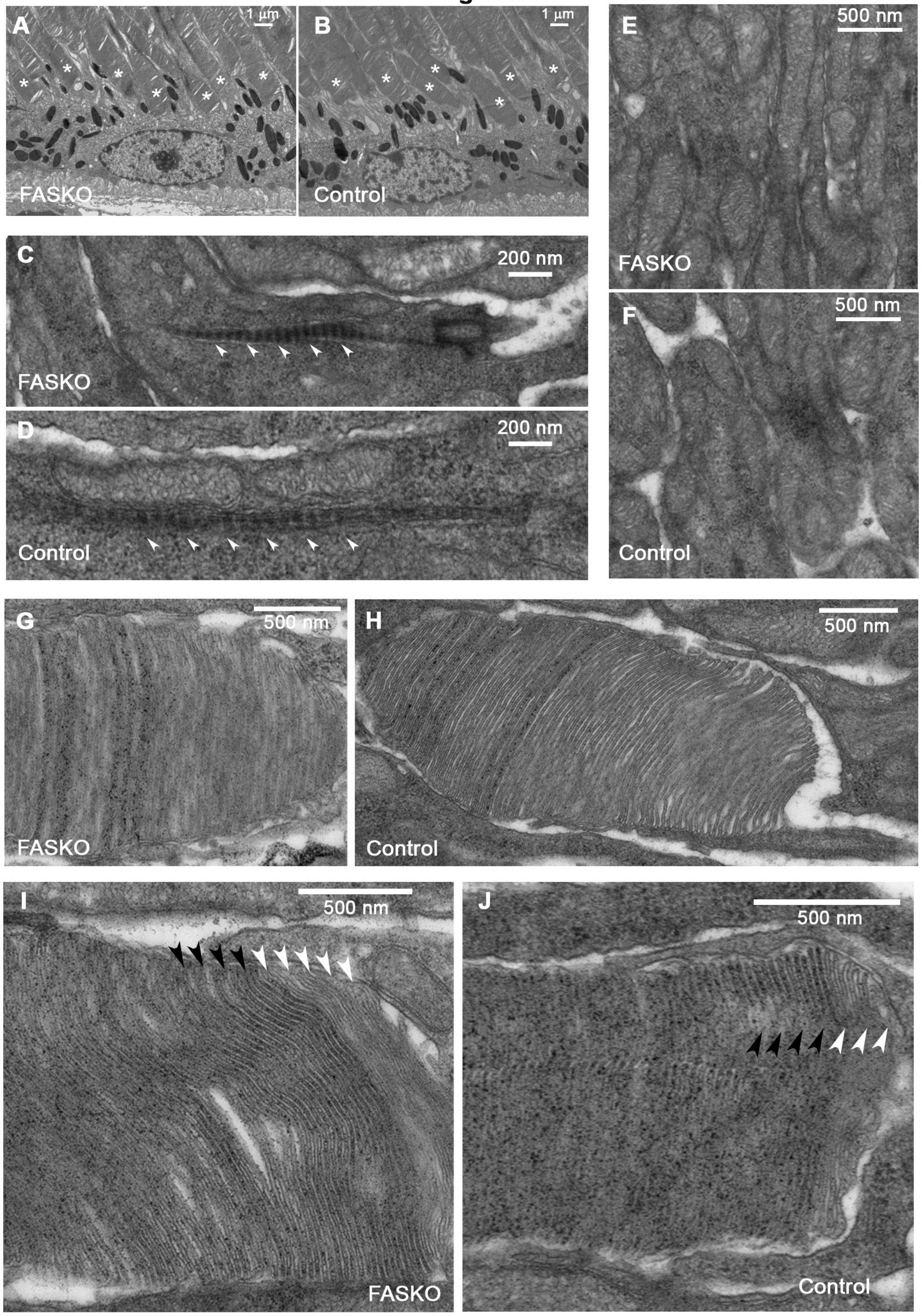

Supplemental Figure 5. Intact ultrastructural morphology in FAS-deficient retina at P28. (**A**, **B**) Transmission electron microscopy demonstrating intact rod photoreceptor outer segment structure (asterisks) and interdigitation with the underlying retinal pigment epithelium in FAS mutants (A) and controls (B). (**C**) Normal appearing rootlet structures in FASKO rods. (**D**) Rootlet structures in controls. (**E**, **F**) Mitochondrial morphology in FASKO rods (E) and littermate control rods (F). (**G**-J) Photoreceptor outer segment detail in FASKO cones (G), control cones (H), FASKO rods (I), and control rods (J). Images are representative of experiments with n=2 for controls and n=5 for FASKO.
